# Similar Allergenicity to Different *Artemisia* Species Is a Consequence of Highly Cross-Reactive Art v 1-Like Molecules

**DOI:** 10.3390/medicina55080504

**Published:** 2019-08-20

**Authors:** Isabel Pablos, Matthias Egger, Eva Vejvar, Victoria Reichl, Peter Briza, Danila Zennaro, Chiara Rafaiani, Winfried Pickl, Barbara Bohle, Adriano Mari, Fatima Ferreira, Gabriele Gadermaier

**Affiliations:** 1 Department of Biosciences, University of Salzburg, 5020 Salzburg, Austria; 2 Institute of Immunology, Center for Pathophysiology, Infection and Immunology, Medical University of Vienna, 1090 Vienna, Austria; 3 Associated Centers for Molecular Allergology, 04100 Rome, Italy; 4 Center for Molecular Allergology, IDI-IRCCS, 00167 Rome, Italy; 5 Institute of Pathophysiology and Allergy Research, Center for Pathophysiology, Infectiology and Immunology, Medical University of Vienna, 1090 Vienna, Austria

**Keywords:** allergens, mugwort pollen, *Artemisia*, Art v 1, IgE cross-reactivity, defensin-like proteins, polyproline-rich protein, allergy, weeds

## Abstract

*Background and objectives:* Pollens of weeds are relevant elicitors of type I allergies. While many *Artemisia* species occur worldwide, allergy research so far has only focused on *Artemisia vulgaris*. We aimed to characterize other prevalent *Artemisia* species regarding their allergen profiles. *Materials and Methods:* Aqueous extracts of pollen from seven *Artemisia* species were characterized by gel electrophoresis and ELISA using sera from mugwort pollen-allergic patients (*n* = 11). The cDNA sequences of defensin–proline-linked proteins (DPLPs) were obtained, and purified proteins were tested in a competition ELISA, in rat basophil mediator release assays, and for activation of Jurkat T cells transduced with an Art v 1-specific TCR. IgE cross-reactivity to other allergens was evaluated using ImmunoCAP and ISAC. *Results:* The protein patterns of *Artemisia* spp. pollen extracts were similar in gel electrophoresis, with a major band at 24 kDa corresponding to DPLPs, like the previously identified Art v 1. Natural Art v 1 potently inhibited IgE binding to immobilized pollen extracts. Six novel Art v 1 homologs with high sequence identity and equivalent IgE reactivity were identified and termed Art ab 1, Art an 1, Art c 1, Art f 1, Art l 1, and Art t 1. All proteins triggered mediator release and cross-reacted at the T cell level. The *Artemisia* extracts contained additional IgE cross-reactive molecules from the nonspecific lipid transfer protein, pectate lyase, profilin, and polcalcin family. *Conclusions:* Our findings demonstrate that DPLPs in various *Artemisia* species have high allergenic potential. Therefore, related *Artemisia* species need to be considered to be allergen elicitors, especially due to the consideration of potential geographic expansion due to climatic changes.

## 1. Introduction

Pollen allergies are one of the most common causes of type I hypersensitivity reactions, affecting up to 30% of the population in industrialized countries. Besides grasses and trees, weeds are considered relevant elicitors of allergic reactions [1]. Weeds are able to grow in ecological niches, as they are highly adaptive and may reproduce in an invasive manner. Due to climatic changes, weeds will be able to spread more easily in barren environments, expand to new geographic areas, and maintain high pollen production [2,3]. As a consequence, increased exposure time and pollen loads could lead to rising sensitization prevalence and incidences of allergic reactions [4,5,6].

The herb mugwort (*Artemisia vulgaris*) belongs to the Asteraceae plant family, which is comprised of about 350 *Artemisia* species. It was originally native to Russia, but is now found across Europe, North America, and parts of Asia. Pollen of *Artemisia* was found to be the predominant species for Asteraceae pollen counts in a study involving 13 pollen monitoring stations across Europe. In Europe, flowering typically starts mid-July and lasts until the end of September (www.pollenwarndienst.at), with a mean duration ranging from 40 to 115 days in Lithuania and Great Britain, respectively [7]. Data from Korea showed a flowering period from July until the end of November, with peak pollen loads in September [8]. In Asia, the plant is frequently used as an edible herb and in traditional Chinese medicine. The geographical distribution of the genus *Artemisia* has been drastically increasing, among other factors due to the commercial cultivation of *Artemisia annua* as a source of artemisinin, a compound identified to possess antimalarial properties and recommended by the World Health Organization as first-line treatment for uncomplicated malaria [9,10]. The areas for crop cultivation are presently dominated by East Asia (China and Vietnam), with recent additions and expansion in East and Southern Africa. Artemisinin is also under investigation for the treatment of infections mediated by viruses, protozoa, helminthes, fungi, cancer, and inflammation [11]. 

*Artemisia vulgaris* represents the best-studied species regarding its allergenic potential and shows a sensitization prevalence of 10%–14% among Central European pollinosis patients [12]. In parts of Asia, allergic reactions to *Artemisia* are particularly relevant and present the highest sensitization frequencies among pollinosis patients, with a reactivity rate of 10.5%–11.3%, while sensitization to grass (3.5%) and tree (2.2%) pollen is comparably low in these geographic regions [13,14]. In a recent study, reactivity to *Artemisia vulgaris* was 58.3% among Chinese patients with confirmed intradermal reactivity to pollen allergens [15]. In Korean patients suffering from respiratory allergies, sensitization to mugwort was reported to be 14.2% [16]. 

The major mugwort pollen allergen Art v 1 belongs to the gamma-thionine family and is a defensin-like protein linked to a polyproline-rich region (defensin–polyproline-linked protein (DPLP)) [17,18,19]. It is a secreted protein with a globular N-terminal cysteine-rich domain and a C-terminal proline-rich region containing several (Ser/Ala)(Pro)_2–4_ repeats. Seven isoallergens of Art v 1 differing in 1–6 residues all located in the C-terminal proline-rich region have been identified [20]. Recombinant production of nonglycosylated allergens in *Escherichia coli* revealed no differences in isoallergens in terms of IgE binding or mediator release potency. Structural integrity and IgE binding capacity were, however, dependent on the cysteine-stabilized fold of the defensin-like domain [17,21]. Epitope mapping of Art v 1 using NMR revealed two distinct structural IgE binding regions located in the defensin-like domain and to a minor extent in the transitional region [22]. 

The natural allergen presents two types of plant-specific O-glycosylations, a large hydroxyproline-linked arabinogalactan and single (but adjacent) β-arabinofuranoses linked to hydroxyprolines, comprising about 30%–40% of molecular mass [23]. While single hydroxyproline-linked β-arabinoses may directly or indirectly comprise IgE binding epitopes, the biological relevance of those glycan-specific antibodies has been shown to be neglectable in mediator release assays (our unpublished data). In contrast to other allergens, Art v 1 presents a single immunodominant T cell epitope, i.e., Art v 1_25–36_, and demonstrates an HLA-DRB1*01 restriction [24,25,26]. Based on this fact, a Jurkat T cell line expressing the human receptor specific for the immunodominant T cell epitope of Art v 1 has been designed, allowing for the study of allergen-specific T cell interactions [27]. Recently, a protective effect for the allergen conjugated to virus-like particles was observed in an Art v 1 mouse model [28,29]. 

Allergenic defensin-like proteins linked to a polyproline-rich region have so far been exclusively found in the pollen of the Asteraceae family. Apart from Art v 1, homologs have been identified in *Ambrosia artemisiifolia* (Amb a 4) and *Parthenium hysterophorus* (Par h 1) [30,31]. Those three members presented varying degrees of IgE cross-reactivity, which mostly depended on the patients’ primary sensitization to the respective pollen source in the different geographic regions [32]. Although the presence of an Art v 1-like molecule in sunflower pollen has been verified using specific antibodies, no further investigations on the allergen (potentially corresponding to anther specific protein SF-18) have been conducted [33]. Further allergenic molecules consisting of a defensin-like domain alone have been described in peanuts and soybeans, but a potential broader implication and connection with other family members remains to be investigated [34,35]. 

Apart from Art v 1, a number of other allergens have been described in *Artemisia* spp. They belong to the protein families of pectate lyases [36,37], nonspecific lipid transfer proteins (nsLTPs) [19,38,39], profilins [40,41], and polcalcins [42] and PR-1 families. Recently, Art an 7, a putative galactose oxidase, was identified and was shown to represent a major allergen in *Artemisia annua* [18,43]. Most of the *Artemisia* allergens described to date have originated from *A. vulgaris*, while other *Artemisia* species have been less investigated.

The aim of this study was to evaluate the allergological relevance of allergenic molecules in *Artemisia* species. Therefore, pollens from seven relevant *Artemisia* species were investigated, and DPLPs were identified as major IgE cross-reactive allergens. Purified DPLPs presented highly similar protein sequences, IgE binding, mediator release, and T cell reactivity in comparison to Art v 1. As a result, six novel allergens were officially acknowledged by the WHO/IUIS Allergen Nomenclature Sub-Committee. The presence of IgE cross-reactive allergens from the pectate lyase, nsLTP, profilin, and polcalcin protein family was demonstrated in the investigated pollen sources. 

## 2. Materials and Methods

### 2.1. Patients’ Sera

Eleven mugwort pollen-allergic patients from Vienna (Austria) with previously determined IgE reactivity to Art v 1 were selected on the basis of typical case histories (recurrent rhinitis/conjunctivitis during late summer), positive skin prick test (≥3 mm) to mugwort pollen extract and allergen-specific IgE (ImmunoCAP ≥ 3) (w6; Phadia, Uppsala, Sweden) [21]. For single-point highest inhibition achievable assays (SPHIAs), a serum pool of five patients with IgE reactivity to Art v 1, nsLTPs, pectate lyase, profilin, and polcalcins, as determined by ImmunoCAP ISAC (Phadia), was used. Experiments with serum samples from patients were approved by the Ethics Committee of the Medical University of Vienna (EK 497/2005) and IDI-IRCCS (106/CE/2005). 

### 2.2. Preparation of Artemisia spp. Pollen Extracts

Pollen grains of *A. absinthium*, *A. annua*, *A. ludoviciana*, and *A. vulgaris* were purchased from Allergon AB (Ängelholm, Sweden); *A. californica*, *A. frigida*, and *A. tridentata* were obtained from Greer Laboratories (Lenoir, NC, USA) (Table 1). Pollen exudates were prepared by rapid agitation in distilled water overnight at 4 °C. Total pollen extracts were obtained by centrifugation and filtered through a 0.45-µm glass fiber filter (Millipore, Billerica, MA, USA). Protein concentrations were determined using a Coomassie Protein Assay Reagent (Thermo Scientific, Rockford, IL, USA), and extracts were stored at −20 °C until further use.

### 2.3. Protein Gel Electrophoresis and Immunoblotting

Protein extracts and purified molecules were separated by reducing gel electrophoresis and visualized using GelCode Blue Safe Protein Stain (Thermo Scientific, Rockford, IL, USA). To analyze interactions with Art v 1-specific antibodies, purified natural proteins were transferred to a nitrocellulose membrane (Whatman, Brentford, UK). Briefly, membranes were incubated with a monoclonal anti-Art v 1 antibody produced by genetic immunization [44] at a dilution of 1:50 in 25 mM Tris/HCl (pH 7.5), 0.5% (w/v) BSA, 150 mM NaCl, 0.5% (v/v) Tween-20, and 0.05% (w/v) NaN_3_. Bound antibodies were detected by rabbit antimouse IgG and IgM alkaline phosphatase conjugates (Jackson Laboratories, West Grove, PA, USA). Detection was performed using nitroblue tetrazolium chloride in combination with 5-bromo-5-chloro-3-indolyl phosphate as a substrate.

### 2.4. Protein Purification

For purification of natural Art v 1 and homologous molecules, total protein extracts were adjusted to 50 mM triethylamine acetate (pH 5.0) and subjected to ion exchange chromatography using the Äkta prime system (GE Healthcare, Chicago, IL, USA) with a 5-mL QFF anion exchange precolumn and a 5-mL CMFF cation exchange column (both GE Healthcare). Fractions containing the target proteins were obtained by gradient elution to 50 mM ammonium bicarbonate (pH 7.9). Proteins were stored at −20 °C.

### 2.5. Mass Spectrometry and N-Terminal Sequencing

Mass spectrometry-based analyses were performed with a Micromass QTof Global Ultima instrument (Waters, Mildford, MA, USA), as previously described [45]. Intact masses were determined by direct infusion; peptides obtained from digestion with trypsin (ProteoExtract Trypsin Digestion Kit, Calbiochem, Gibbstown, NJ, USA) or subtilisin (Sigma-Aldrich, St. Louis, MO, USA) were separated by capillary HPLC online coupled to a mass spectrometer. For de novo sequencing, the ProteinLynx Global Server, software version 2.2.5 (Waters), was used. N-terminal sequencing by Edman degradation was done by the protein sequencing service of the Division of Clinical Biochemistry at the University of Innsbruck, Austria. 

### 2.6. cDNA Cloning

Total RNA from *Artemisia* spp. pollen was prepared using TRIzol LS Reagent (Invitrogen, Carlsbad, CA, USA) according to the manufacturer’s protocol. Briefly, pollen grains were ground with glass sand under liquid nitrogen. Pollen RNA was extracted by the addition of TRIzol and chloroform and precipitated from the aqueous phase. Full-length DNA sequences of Art v 1 homologs were obtained by reverse transcription from cDNA using a SuperScriptTM One-Step RT-PCR with a Platinum Taq kit (Invitrogen, Carlsbad, CA, USA) with specific 5′-GGAATTCCATATGGCTGGTTCAAAGTTGTGTGAAAAG-3′ forward and 5′-GAGAGAATTC(T)_21_-3′ reverse primer. In addition, cDNA and protein sequences were aligned and analyzed using the online tool ClustalW2 (http://www.ebi.ac.uk/clustalw2). 

### 2.7. ELISA and Cross-Inhibition Studies

Maxisorp plates (Nalge Nunc, Rochester, NY, USA) were coated with 200 ng of pollen extracts or purified molecules in PBS overnight at 4 °C. Unspecific binding was blocked with TBS (pH 7.4), 0.05% (v/v) Tween, and 1% (w/v) BSA and incubated with patients’ sera (1:4) overnight at 4 °C. Bound IgE was detected with alkaline phosphatase-conjugated monoclonal antihuman IgE antibodies (BD Biosciences, Franklin Laker, NJ, USA). In addition, 4-nitrophenylphosphate (Sigma-Aldrich, St. Louis, MO, USA) was used as a substrate, and optical density was measured at 405/492 nm. Values were considered positive when exceeding 3 times the standard deviation of buffer control vials. For inhibition experiments, patients’ sera were pre-incubated overnight at 4 °C with increasing concentrations (0.005–5 µg/mL) of purified natural Art v 1.

### 2.8. Mediator Release Assay

Mediator release of purified DPLPs from *Artemisia* spp. was evaluated using a rat basophil degranulation leukemia (RBL) assay, as described in Reference [21]. RBL-2H3 cells, which were transfected with an antihuman IgE high-affinity receptor, were sensitized with patients’ sera overnight (*n* = 4). Cells were stimulated with increasing concentrations of the purified proteins (0.001–100 µg/mL), and the release of β-hexosaminidase was measured by the enzymatic cleavage of the fluorogenic substrate 4-methylumbelliferyl *N*-acetyl-β-D-glucosaminide (Sigma, Germany). Total release (100%) was obtained when cells were treated with 10% of Triton X-100, and mediator release of DPLPs is given as a percentage.

### 2.9. T Cell Reactivity

Art v 1 TCR transgenic (tg) Jurkat T cells expressing an IL-2 enhancer/promoter-driving luciferase were cultured as described [27]. Antigen-presenting 293 cells (1.5 × 10^6^) were generated by transient cotransfection with HLA-DRA*01:01, HLA-DRB1*01:01, CD80::GPI, CD54::GPI, and cathepsin S (total of 30 µg DNA/10 cm dish) and then pre-incubated at 5 × 10^4^/well in 96-well flat-bottom culture plates with indicated concentrations of purified DPLP proteins (range: 6–0.01 × 10^−6^ M), Art v 1_25–34_ peptides (range: 96–0.01 × 10^−6^ M), or medium alone in a total volume of 100 µl for two hours. 

Subsequently, TCRtg Jurkat cells (1 × 10^5^) were added to the artificial APCs and cocultured for 6 h. PMA (10^−7^ M) plus PHA (5 µg/mL) served as a positive control, and medium alone was a negative control. Cells were lysed, and luciferase activity was determined (Promega, Madison, WI, USA) on an Infinite M200 luminometer (Tecan, Männedorf, Switzerland).

### 2.10. ISAC Inhibition Studies

Single-point highest inhibition achievable assays (SPHIAs) were performed, as previously reported [39]. Briefly, sera from mugwort-allergic patients (*n* = 5) with specific IgE to Art v 1 and other relevant sensitizations were pooled. Fifteen microliters of sera were pre-incubated with an equal volume of *Artemisia* spp. pollen extract (0.5 mg/mL) and incubated overnight. IgE binding to Art v 1, nsLTPs (Art v 3, Cor a 8, and Pru p 3), pectate lyases (Amb a 1, Cry j 1, and Cup a 1), profilins (Bet v 2, Phl p 12, Hev b 8, Ole e 2, and Mer a 1) and polcalcins (Bet v 4 and Phl p 7) on an ISAC 103 microarray (PMD, Vienna, Austria) were evaluated, comparing samples incubated with pollen extracts and buffer as a control. Results are reported as percentage of IgE inhibition. Nonspecific IgE inhibition was evaluated by results obtained from other nonrelevant allergens.

### 2.11. Statistics

ELISA analysis of *Artemisia* spp. extracts was performed using a one-way ANOVA and Tukey’s multiple comparisons test. An analysis of purified DPLPs was conducted using the Friedman test and Dunn’s multiple comparison test: *p*-values < 0.05 were considered statistically significant.

## 3. Results

### 3.1. Geographic Distribution of Artemisia *spp.* and Analysis of Pollen Proteins 

**Figure 1:**
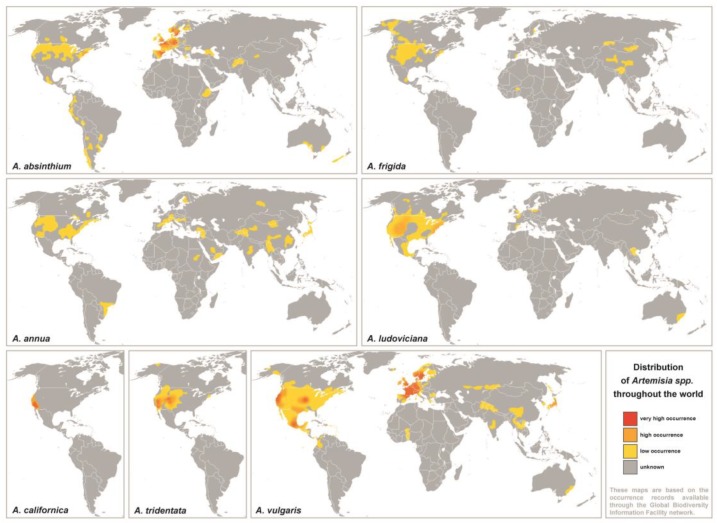
Worldwide distribution of investigated *Artemisia* species. The maps are based on occurrence records available through the Global Biodiversity Information Facility network (http://data.gbif.org). Data on distributions of *A. californica* and *A. tridentata* were restricted to North America.

**Figure 2:**
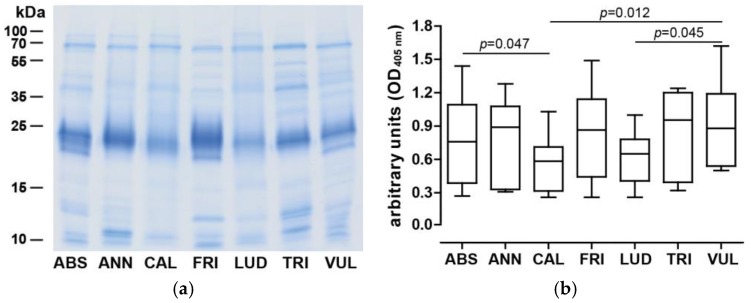
Protein composition and IgE reactivity of *Artemisia* spp. pollen extracts. (**a**) Protein extracts (8 µg per lane) were separated by denaturing gel electrophoresis; (**b**) IgE reactivity to *Artemisia* extracts was evaluated in an ELISA using sera of mugwort pollen-allergic patients (*n* = 11). Boxes indicate the interquartile ranges, the median is shown as a solid line, and whiskers represent the 5th and 95th percentile. One-way ANOVA using Tukey’s post hoc test was used for statistical analysis. ABS, *A. absinthium*; ANN, *A. annua*; CAL, *A. californica*; FRI, *A. frigida*; LUD, *A. ludoviciana*; TRI, *A. tridentata*; VUL, *A. vulgaris.*

A panel of seven representative *Artemisia* species differently spread throughout the world was selected for this study (Table 1, Figure 1). Aqueous extracts of all investigated species showed similar protein patterns in denaturing gel electrophoresis (Figure 2a). A protein migrating at around 24 kDa was predominant in all *Artemisia* spp. pollen extracts. A characteristic two-band migration pattern previously determined for Art v 1 was also observed for the corresponding proteins in *A. absinthium* and *A. frigida*, while other extracts presented only a single band. In the lower molecular weight range, various different protein patterns were found, most likely representing profilins, nsLTPs, and polcalcins. 

### 3.2. IgE Binding Capacity and Art v 1 Cross-Reactivity Pattern of Artemisia Extracts

Sera of 11 Austrian mugwort pollen-allergic patients were used to determine IgE reactivity toward different *Artemisia spp.* pollen extracts (Figure 2b). In general, similar IgE reactivity patterns were observed, while *A. californica* and *A. ludoviciana* presented significantly lower values. 

To assess the presence of Art v 1-like molecules, competition ELISA with five sera was performed using purified nArt v 1 as an inhibitory molecule (Table 2). Similar dose-dependent inhibitions were noted for all tested pollen extracts, reaching 82.1%–90.5% when the highest inhibitor concentration was used. However, inhibition was already high when lower concentrations of 0.005 µg/mL were used, while there were variations between patients, indicated by a higher standard deviation at this concentration. In summary, the presence of IgE cross-reactive DPLPs was unambiguously demonstrated. 

### 3.3. Purification of DPLPs

**Figure 3:**
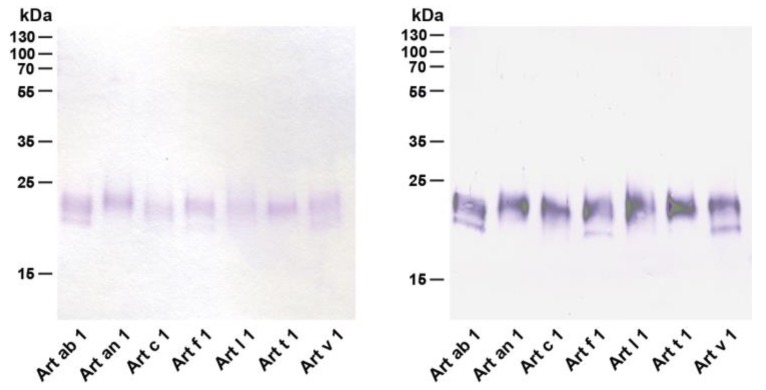
Quality assessment of purified natural defensin–proline-linked proteins (DPLPs). Purified proteins were monitored by denaturing gel electrophoresis (left) and immunoblot analysis using a monoclonal anti-Art v 1 antibody [44] (right).

DPLPs were purified to homogeneity from aqueous pollen extracts, and a purity of >95% was assessed by denaturing gel electrophoresis. A similar two-band migration pattern that had been observed earlier in pollen extracts was apparent for Art ab 1, Art f 1, and Art v 1 while other homologs presented one band (Figure 3, left panel). All purified allergens were recognized by a monoclonal anti-Art v 1 antibody [44] in immunoblot experiments. The antibody recognized the upper and lower protein band (if present) at equivalent intensities corresponding to the quantity in gel electrophoresis (Figure 3, right panel). 

### 3.4. cDNA Cloning and Amino Acid Sequence Identification 

**Figure 4:**
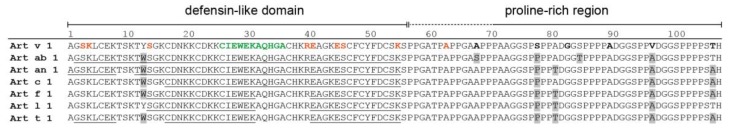
Sequence alignment of DPLPs. Amino acids differing from Art v 1.0101 are depicted in gray boxes. Underlined sequences in the defensin-like domain correspond to peptides identified by mass spectrometry. Residues in the sequence of Art v 1.0101 previously identified to be different in other isoforms [20] are shown in bold, and the T cell and IgE binding epitopes are indicated in green [26] and orange [22], respectively. The transitional region between the defensin-like and proline-rich domain is depicted as a dotted line.

N-terminal sequencing revealed that processed N-termini of investigated mature proteins were identical (AGSKLCEKTS). Thus, a forward primer corresponding to the first eight amino acids of the mature protein was used to obtain the cDNA sequence. As allergenicity of the molecules was subsequently demonstrated, they were officially acknowledged by the WHO/IUIS allergen nomenclature subcommittee with the following allergen names (GenBank entries in brackets): Art ab 1 (KC700032.1), Art an 1 (KC700033.1), Art c 1 (KC700034.1), Art f 1 (KC700035.1), Art l 1 (KC700036.1), and Art t 1 (KC700037.1). Obtained clones showed highly similar DNA sequences, with identities ranging from 95.4% to 100% (Art an 1 and Art f 1 presented identical cDNA sequences). Those sequences translated into amino acid sequences of at least 95.4% identity and seven residues that were subject to variations in comparison to Art v 1.0101. Identical protein sequences were obtained for the DPLPs from *A. annua*, *A. frigida*, and *A. tridentate.* Differences were mostly confined to the polyproline-rich domain (Figure 4). Interestingly, one position in the defensin-like domain seemed to be consistently different (tryptophan at residue 13) in most of the investigated homologous sequences. Only the sequence of Art l 1 showed a tyrosine residue at this position, analogous to Art v 1. Trypsin and subtilisin peptide fragments analyzed by mass spectrometry were able to confirm 60% to 95% of the sequence in the defensin-like domain. Due to a lack of proteolytic cleavage sites in the polyproline-rich domain, peptide-based mass spectrometry of the C-terminal region was not feasible.

### 3.5. IgE Reactivity to Purified DPLPs

**Figure 5:**
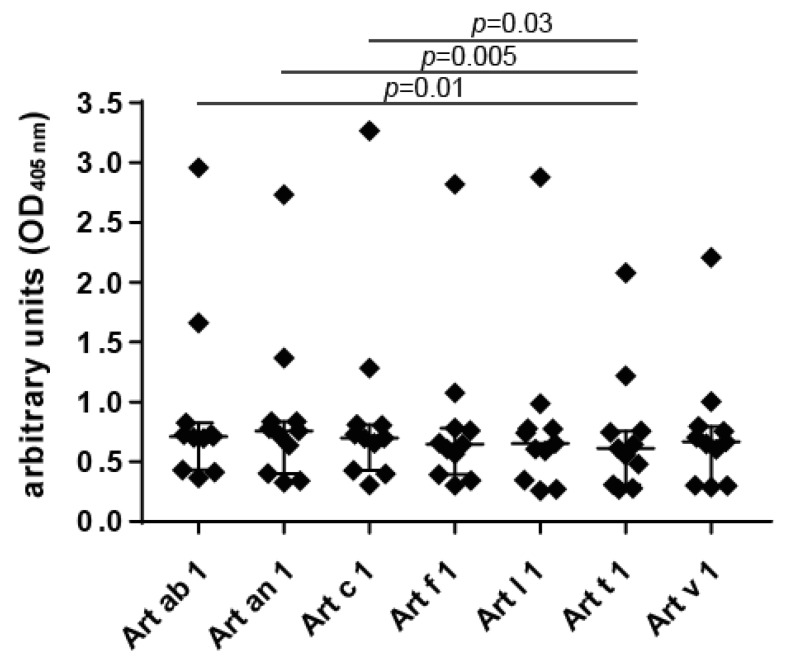
IgE reactivity to purified allergens investigated in an ELISA using mugwort pollen-allergic patients’ sera (*n* = 11). The median is shown as a solid line, and whiskers represent the interquartile range. Statistical evaluation was performed using the Friedman test followed by a post hoc Dunn’s multiple comparisons test.

The allergological relevance of the DPLPs purified from *Artemisia* species was evaluated in an ELISA using the sera of 11 mugwort pollen-allergic patients sensitized to Art v 1 (Figure 5). All homologous molecules were recognized by patients’ IgE in a similar manner. Art t 1 presented with significantly lower IgE reactivity compared to Art ab 1, Art a 1, and Art c 1. Slightly higher reactivity compared to Art v 1 was observed with some DPLP representatives, which was, however, beyond statistical significance. 

### 3.6. Mediator Release Assay

**Figure 6:**
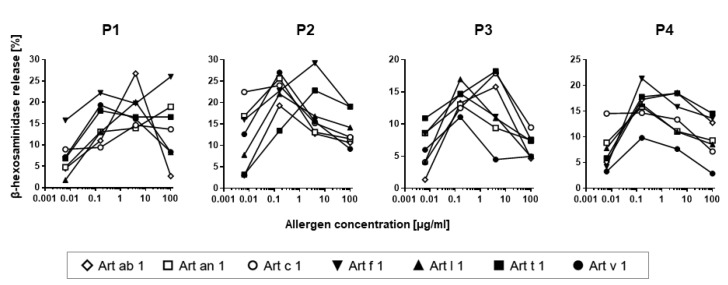
Mediator release assay. Rat basophil leukemia (RBL) cells transfected with an anti-human IgE high-affinity receptor (RBL-2H3) were sensitized with four mugwort-allergic patients’ sera (**P1**–**P4**). Cells were stimulated with increasing concentrations of purified allergens, and the release of β-hexosaminidase was measured and is expressed as a percentage of total release with Triton X-100.

To verify the biological activity of the homologous DPLPs, the mediator release of rat basophil leukemia cells with an antihuman IgE high-affinity receptor was performed. Four sera with strong IgE reactivity to Art v 1 were selected, and β-hexosaminidase release triggered by different concentrations of purified allergens was measured (Figure 6). All purified DPLPs presented with dose-dependent mediator release curves, and the highest release was observed at a 0.16–4.0 µg/mL allergen concentration. Depending on the patients tested, homologous molecules were able to trigger similar but also higher mediator release compared to Art v 1. All purified DPLPs from *Artemisia* spp. presented with the ability to efficiently cross-link receptor-bound IgE antibodies. 

### 3.7. Art v 1 and DPLPs Presented T cell Cross-Reactivity

**Figure 7:**
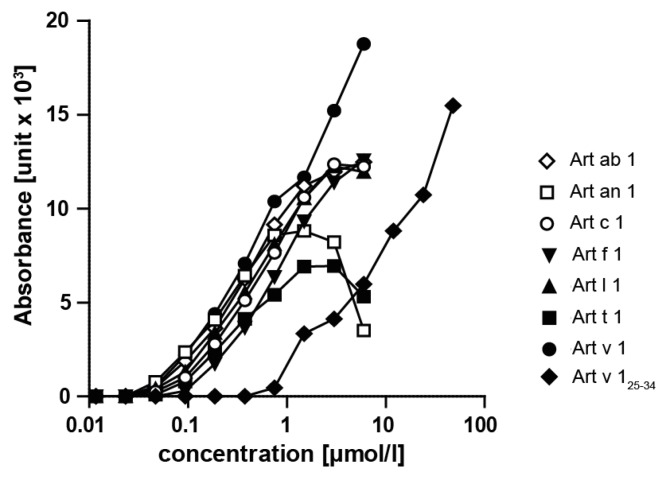
The Art v 1_25–36_-specific TCR cross-reacted with purified DPLP proteins presented on APCs. HLA-DR1^+^ artificial APCs were pre-incubated for 2 h with purified DPLPs, and Art v 1_25–34_ peptides were cocultured with TCRtg Jurkat cells for 6 h before luciferase activity was determined. Data show mean values of triplicate cultures representative of two independently performed experiments.

To evaluate the cross-reactivity at the T cell level, Jurkat T cells transduced with an Art v 1_25–34_ TCR (recognizing the immunodominant T cell epitope of Art v 1) were used [27]. Using different concentrations of purified DPLPs as well as the Art v 1_25–34_ peptide, dose-related responses were observed (Figure 7). In a concentration range up to 1 µmol/L, similar curves were observed for all purified allergens. Art v 1 triggered elevated responses, while Art an 1 and Art t 1 presented a lower absorbance in a higher concentration range. The Art v 1_25–34_ peptide required around 100-fold higher concentrations to trigger a similar response. PMA plus PHA stimulation served as an independent positive control, and medium alone was a negative control. 

### 3.8. IgE Cross-Reactivity within Other Allergenic Protein Families

**Figure 8:**
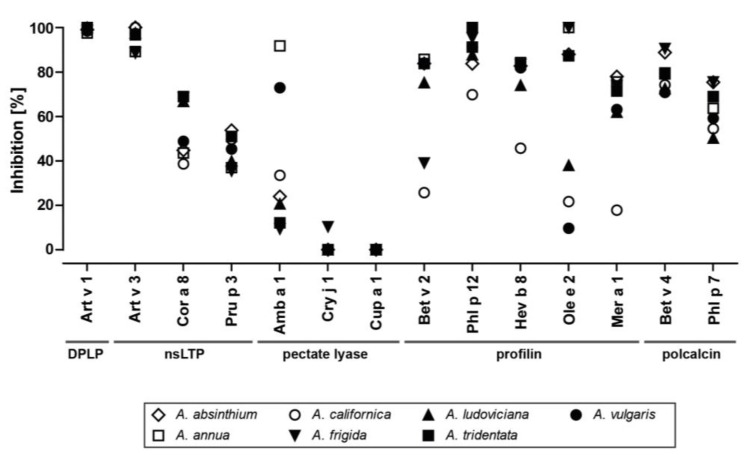
IgE cross-inhibition to ImmunoCAP ISAC allergens using different *Artemisia* species. Single-point highest inhibition achievable assays (SPHIAs) were performed using a serum pool of mugwort pollen-allergic patients.

To evaluate the relevance of allergens belonging to families other than the DPLP family, ISAC inhibition studies were performed (Figure 8). Therefore, total protein extracts of *Artemisia* species were used to inhibit serum IgE binding to allergens immobilized on an ImmunoCAP ISAC (single-point highest inhibition achievable assays (SPHIAs)). IgE reactivity to Art v 1 was entirely inhibited by all *Artemisia* spp. extracts. In addition, very high IgE cross-inhibition to Art v 3, the nsLTP of *A. vulgaris*, was observed, and to a lesser extent to representatives from hazelnut (Cor a 8) and peach (Pru p 3). IgE cross-inhibition to the pectate lyase Amb a 1 was high in *A. annua* and *A. vulgaris*, but only moderate regarding residual pollen extracts. Negligible cross-reactivity with pectate lyses from Japanese cedar (Cry j 1) and cypress (Cup a 1) was noticed. Regarding the panallergens profilin and polcalcin, diverse patterns were observed, ranging from high IgE cross-reactivity to moderate/low cross-reactivity, especially when *A. californica* extracts were used as an inhibitor. 

## 4. Discussion

Pollens from weeds represent important contributors to allergic reactions. The Asteraceae plant family comprises relevant allergenic plants such as mugwort, ragweed, feverfew, and sunflower. So far, molecular allergology in the various *Artemisia* species has focused on *A. vulgaris*, which grows in temperate climate zones and is known to cause allergic reactions in late summer and autumn. In this study, we extended our allergological research to additional species, i.e., *A. absinthium* (also present in South America), *A. frigida* (also found in parts of Alaska), *A. annua* (cultivated for artemisinin production), *A. californica*, and *A. tridentata* (specific occurrence in some parts of the United States). For comparison and due to the fact that our patient cohort was primarily sensitized to *A. vulgaris*, its pollen was included as a reference. Those representatives are, e.g., termed mugwort, wormwood, and sagebrush, and several are used as herbal medicines and for treatments for malaria [9,10]. *Artemisia* are present in many regions of the world, and due to climatic changes, expansion to geographic regions beyond the current habitats is expected [2,3]. 

We found that aqueous protein extracts of *Artemisia* pollen presented similar migration patterns in gel electrophoresis, with a dominant band migrating around 24 kDa (corresponding to DPLPs). Previously, seven Art v 1 isoallergens were identified, which contribute to different proline and thus varying O-glycosylation patterns [20,23]. Consequently, Art v 1 appeared as two bands in gel electrophoresis, with heterogeneous masses ranging from 12.9–13.5 and 14.1–16.3 kDa [17]. *A. absinthium* and *A. frigida* presented analogous DPLP patterns, while others localized in one band. Representatives of this allergen family seem to generally constitute highly diverse glycosylation patterns [23,30], and further investigation is required to unravel the exact positions of the hydroxyproline-linked glycan moieties.

Using sera from mugwort pollen-allergic patients, similar IgE reactivity was observed with slightly lower reaction toward *A. californica* and *A. ludoviciana*, which corresponded to a less prominent appearance of the DPLPs in gel electrophoresis. In addition, there was a lack of protein bands in the lower molecular weight region where panallergens are typically detectable. Results from the SPHIA experiments showed a very low IgE inhibition of *A. californica* toward profilin, indicating a substantially lower amount of this allergen in this extract. Even though extraction protocols were consistent for all pollens and equivalent protein amounts were loaded on the gel, there was a certain degree of variation between the extracts. This is explainable by the different environmental conditions those plants were exposed to. Fluctuation levels of allergens can be explained by the fact that many of them are pathogenesis-related proteins that directly respond to external (stress) factors. This also highlights (again) why the standardization of allergen extracts is a challenging task. To verify the presence of a DPLP, we performed IgE inhibition ELISAs using purified natural Art v 1. Indeed, we observed very high IgE inhibitions up to 90.5%. Interestingly, inhibition against *A. vulgaris* did not achieve the highest levels, which was explained by further IgE reactivity to additional mugwort pollen allergens, which were not inhibited by Art v 1. The results, however, clearly indicate that Art v 1-homologous molecules represented major allergens in all of the investigated pollen sources.

To this end, we chromatographically purified DPLPs from seven *Artemisia* species, which were detected by a monoclonal anti-Art v 1 antibody [44]. As this antibody was directed against the defensin-like domain, all apparent bands, although differing in their glycan profiles, were recognized in an analogous manner [20]. As investigated allergens presented with the same N-terminal residues, cDNA cloning using an Art v 1-specific primer was pursued. Very high sequence identities at the DNA as well as the protein level were observed. Amino acid variations were mostly confined to the polyproline-rich region and involved residues also varying in the isoallergens of Art v 1 [20]. All proteins except Art v 1 and Art l 1 presented a tryptophan residue at position 13. Interestingly, the DPLPs of ragweed, feverfews, and sunflowers also demonstrated a tryptophan at this position, while the sequence in the polyproline-rich region was highly divergent from all *Artemisia* representatives [30,31]. Using mass spectrometry-based analyses, we were able to cover large parts of the sequence of the defensin-like domain. The C-terminal part could not be assigned due to a lack of proteolytic cleavage sites for suitable peptide generation and high heterogeneity due to glycosylation and potentially isoallergens.

We further assessed allergen binding and mediator release capacities using the sera of Art v 1-sensitized mugwort pollen-allergic patients. Generally, highly similar IgE binding capacities were observed, and all allergens were able to trigger a significant β-hexosaminidase release, corroborating functional activity at individual and patient-specific release levels. While in the ELISAs reduced IgE binding toward Art t 1 was noted, this interestingly did not translate to lower mediator release capacity in the four tested patients’ sera. The presence of tryptophan or tyrosine at residue 13 did not lead to any significant differences in the IgE binding or mediator release patterns. In fact, the residues shown to be directly involved in antibody binding were S3, K4, S16, R40, E41, E45, S46, K55, and A63, and all of those were conserved in all newly identified sequences. However, glycan moieties might play a role in the Art v 1 fold, as the transitional region in the polyproline-rich region can interact with the defensin-like domain [22]. This might in turn also prevent some of the IgE antibodies from binding and would explain variations in the recognition pattern. Varying degrees of mediator release were also notable for DPLPs, which did not, however, follow a trend, but were patient-specific, suggesting the recognition of different surface-located IgE epitopes. 

To assess T cell cross-reactivity, we used Jurkat T cells with a receptor specific for the single, immunodominant T cell epitope of Art v 1, located in the sequence Art v 1_25–34_ [26,27]. Identified cDNA sequences of DPLPs presented identical residues at this relevant T cell-activating region. The fact that purified Art v 1 revealed a stronger reaction while Art an 1 and Art t 1 triggered lower responses in the higher concentrations tested was therefore unexpected. This was potentially due to variations in the C-terminus, i.e., in glycosylation patterns. Variations observed only in the higher concentration ranges suggest differences in kinetics regarding antigen uptake and/or processing. However, a high degree of T cell cross-reactivity was revealed for all homologous allergens. It is noteworthy to mention that the interaction with the receptor was very specific, as Amb a 4 and Par h 1, which presented very similar residues in the corresponding region, did not trigger any T cell responses in a previous study [32].

Based on the results obtained with the DPLPs, we evaluated the presence of other IgE reactive molecules in the selected *Artemisia* species. For this, we used a serum pool of mugwort pollen-allergic patients with broad allergen recognition and evaluated IgE cross-reactivity using all seven extracts [39,46]. The results clearly demonstrated the presence of Art v 1 cross-reactive molecules, and we confirmed the above results with another assay and patients’ sera. High IgE cross-reactivity to Art v 3 and intermediate cross-reactivity to Pru p 3 and Cor a 8 from peaches and hazelnuts, respectively, were found [39]. Diverse IgE inhibition ranging from 12% to 92 % was detected toward the pectate lyase Amb a 1 from ragweed pollen, potentially involving Art v 6 from mugwort pollen and other *Artemisia* pectate lyases [37,47]. It seems that different protein amounts or isoform distributions of the homologous pectate lyases accounted for the observed results [48]. In contrast, no cross-inhibition was observed for the pectate lyases from Japanese cedar and cypress, which was expected based on the low sequence conservation [37]. High IgE cross-reactivity with profilin and polcalcin and lower inhibitory capacity of *A. californica* could be explained by a low quantity of panallergens, as was observed in the gel electrophoresis (around 14 kDa). In summary, we confirmed the presence of IgE cross-reactive molecules from the DPLP, nsLTP, pectate lyase, profilin, and polcalcin family. The results also demonstrated heterogeneity in terms of protein and allergen amounts. We thus propose the use of purified allergen molecules for diagnosis, as quality and quantity can be standardized [49].

## 5. Conclusions

This study demonstrated the high allergological and immunological similarity of DPLPs in pollen from seven *Artemisia* species. Thus, exposure and clinical relevance go beyond the primarily investigated species *A. vulgaris*. Because of climatic changes and further expansions of weed habitats, an increase in sensitization and symptoms due to *Artemisia* spp. pollen may be encountered. 

## Figures and Tables

**Table 1:** List of *Artemisia* spp. investigated in this study.

Latin Name	Common Name	Special Use
*A. absinthium*	absinthe wormwood, wormwood, green ginger, or grand wormwood	medicinal herb, ingredient of the liqueur “absinthe”
*A. annua*	sweet wormwood, sweet sage wort, or annual wormwood	traditional Chinese herbal medicine; leaves are used for antimalaria treatment (artemisinin)
*A. californica*	California sagebrush	spices and tea
*A. frigida*	sagebrush, prairie sage wort, or fringed wormwood	medicinal herb containing camphor
*A. ludoviciana*	silver wormwood or white sagebrush	medicinal herb
*A. tridentata*	common sagebrush or mountain sagebrush	traditional medicine of North American Indian tribes
*A. vulgaris*	mugwort or common wormwood	traditional Chinese herbal medicine

Special uses were acquired from Plants for A Future (http://www.pfaf.org).

**Table 2:** IgE inhibition to *Artemisia* spp. using purified natural Art v 1.

	% MI (SD) 0.005 µg/mL nArt v 1	% MI (SD) 0.05 µg/mL nArt v 1	% MI (SD) 0.5 µg/mL nArt v 1	% MI (SD) 5.0 µg/mL nArt v 1
***A. absinthium***	70.4 (20.4)	75.8 (17.9)	80.5 (11.7)	85.3 (6.8)
***A. annua***	75.2 (17.5)	81.5 (16.3)	85.2 (9.3)	90.2 (5.8)
***A. californica***	72.8 (15.6)	80.0 (15.1)	90.5 (8.4)	90.5 (5.2)
***A. frigida***	74.3 (16.8)	82.5 (12.7)	86.7 (7.2)	90.2 (5.4)
***A. ludoviciana***	69.5 (21.2)	75.6 (19.4)	81.7 (10.3)	87.3 (6.2)
***A. tridentata***	73.9 (17.0)	80.0 (14.9)	84.1 (10.9)	87.2 (8.0)
***A. vulgaris***	63.8 (21.2)	76.4 (17.9)	78.6 (10.6)	82.1 (6.6)

MI, mean inhibition; SD, standard deviation.
